# Towards an integrative self: a digital photo elicitation study of resilience among key marginalized populations of sexual and gender minority youth

**DOI:** 10.1080/17482631.2021.1961572

**Published:** 2021-08-10

**Authors:** Shelley L. Craig, Andrew D. Eaton, Alexa Kirkland, Egag Egag, Rachael Pascoe, Kourteney King, Sreedevi Krishnan

**Affiliations:** a Factor-Inwentash Faculty of Social Work, University of Toronto, Toronto, ON, Canada; b Faculty of Social Work, University of Regina – Saskatoon Campus, Regina, Canada; c Ontario Institute for Studies in Education, University of Toronto, Toronto, ON, Canada

**Keywords:** LGBTQ youth, digital methods, photo elicitation, grounded theory, online research, Canada

## Abstract

Purpose: Sexual and gender minority youth (SGMY) experience unique challenges related to identity and disclosure, and cope in vibrant ways. Qualitative research has not yet fulsomely explored the risk, resilience, and identity intersections that impact vulnerable SGMY wellbeing. Methods: This digital photo-elicitation study (QueerView) recruited thirty SGMY (aged 14–29) from priority populations that had one or more of the following experiences:  trans and gender diverse, homelessness, child welfare, and immigration. From submission of fifteen photos representing resilience and a semi-structured interview via web conferencing, constructivist grounded theory was utilized for multimodal analysis of photos, interview video, and interview transcript. Triangulation, an audit trail, and member checking were employed to support trustworthiness. Results: A visual model emerged showing how participants work towards an integrative self, with themes of reflecting and knowing, discrimination and intersectional challenges, connecting, performing, curating, coping, (re)defining and (re)creating, growing and being. Sub-themes of the impact of family dynamic and values, mental health and trauma, and the cathartic benefit from advocacy and leadership offered insight. Participant images were captured in a digital gallery. Conclusions: QueerView animates the complex lives of multiply marginalized SGMY and their intersectional strengths and challenges while demonstrating the utility of a digital multimodal approach.

Digital technologies often provide a space for sexual and gender minority youth (SGMY) whose sexual orientation, gender identities, and expressions are considered non-majority (not cisgender, heterosexual); including but not limited to lesbian, gay, bisexual, transgender, queer, and non-binary (Craig et al., 2017b), to engage with others and develop their identities (Marwick et al., 2010). SGMY report that these online communities allow them to build confidence, hope, and belonging, which ultimately drive a desire to live (Austin et al., 2020). Such experiences are critical, given that SGMY experience heightened adversity and oppression during their adolescence and young adulthood compared to their heterosexual, cisgender peers (Berghe et al., 2010). Such experiences contribute to social isolation, mental health issues, substance use, and suicidality (McDonald, 2018), which have lasting implications on their physical, emotional, and social selves (Craig & Austin, 2016). SGMY’s negative health and mental health outcomes (e.g., depression and suicidality) are attributed to minority stress—the internal and external stressors that are specific to those with a sexual or gender identity that differs from common societal expectations (Meyer, 2003). These stressors can manifest in institutional and individual forms such as public policy, internalized stigma, microaggressions, transphobia, and violence. Early work about minority stress explores the relationship between sexual minority identities in relation to other identities such as gender and socioeconomic status (Brooks, 1981; Rich et al., 2020) and the differences in stress appraisal across individuals, which is often dependent on their coping resources. Meyer (2015) describes resilience for sexual minority youth as a process of experiencing these stressors, both distal (external) and proximal (internal) through harnessing their coping skills in order to positively impact their mental and physical health. The utilization of digital technologies and social media has been found to contribute to the resilience of SGMY even in the face of traumatic events (Craig et al., 2020a) and is considered safer than offline activity due to the greater ability to produce, manage, and remain in control of their identities and overall comfort online (Craig et al., 2015). Given these experiences, digital research methods are accessible, appropriate and potentially preferable to capture the lived experiences of SGMY.

## Intersectionality

Coined by Crenshaw, intersectionality refers to the intersection of identities such as gender and race and how they can influence personal experiences within various structures in the world (1991). Given that SGMY represent a diverse collective of youth with various sexual orientations, gender identities and expressions, and that their experiences as SGMY may be influenced by various identities related to other forms of oppression (i.e., race, ethnicity, disability, and poverty), employing an intersectionality framework allows for a nuanced exploration of their lived experiences. The term “intersectionality” is not all-encompassing with one specific interpretation (Collins, 2012) as both oppression and privilege exist within one’s intersectional identities, which may be fluid over time and influenced by the political, environmental, and personal context (Hulko, 2009). Using an intersectional lens serves to explore how all identities interact with one another (Hulko & Hovanes, 2018) and allows for an analysis of power when conducting research with SGMY (Wagaman, 2014). Intersectionality research with SGMY encourages exploration of the ways in which their LGBTQ+ identity interacts with oppressed and privileged identities (Sterzing et al., 2017) and allows for more effective interventions that helping professionals can utilize to meet the diverse needs of SGMY (Huang et al., 2020).

There are some common concepts that are applied while using intersectionality theory in qualitative research: a) that all participant identities consist of privilege, power, and oppression interlinked; b) that each identity intersects with every other; and c) that these identities are shaped by the socio-cultural context in which the participant lives (Else-Quest & Hyde, 2016). Intersectionality theory within qualitative research extends beyond methodology and can be consistently applied throughout different stages of the research process (Abrams et al., 2020). Rosenthal (2016) advises that researchers be careful not to simplify their use of intersectionality theory, but rather address responses to adversity that are shaped by structural and societal inequities, such as participants’ strengths and resilience.

## Resilience

Resilience is considered a social ecological construct (Luthar et al., 2000; Ungar, 2011) which describes how people utilize internal skills and coping strategies in order to thrive and overcome adversities and trauma (Masten & Powell, 2003; Ungar, 2008). From a holistic perspective, and in relation to LGBTQ+ youth research, resilience can be defined as an integrated process that does not occur in isolation within an individual, but rather across external systems (Colpitts & Gahagan, 2016). Despite the oppression and violence SGMY experience within families, schools, and their broader communities, they constantly adjust their coping mechanisms to promote resilience and overall wellbeing (DiFulvio, 2015). Contributors to resilience in SGMY include social connectedness, self-efficacy, and healthy coping as they form supportive and empowering relationships where their identities are valued (Amodeo et al., 2018; Craig et al., 2015). Research using minority stress and intersectionality theory has noted that stress encountered by SGMY with multiple marginalization is challenging but also provides an opportunity for developing resilience (Craig et al., 2017a; Meyer, 2010). Such post-traumatic growth is frequently witnessed in research with SGMY (Joseph et al., 2012). For example, an investigation of Latinx/a SGMY found that youth leveraged those intersections in ways that supported their own and their families’ understanding and reflection of familial expectations and supports (Craig et al., 2017a). Resilience studies are compatible with intersectionality when they consider the individual’s social location, acknowledge the interaction between community and individual level attributes in analysing their navigation of adversity, and account for access to social resources (Bowling et al., 2019). Before engaging with resilience frameworks within qualitative research, it is crucial that researchers challenge themselves to openly accept the narratives while also analysing the resilience processes that may not be explicitly stated by participants (Liebenberg et al., 2014).

## Priority populations of SGMY

Most research has either explored the common experiences across all SGMY identities (Craig et al., 2017b) or small subpopulations (Goffnett & Paceley, 2020; McCormick & Krieger, 2020) with few studies focusing on the intersections of SGMY identities and experiences. In consultation with community advisors that identified at-risk subpopulations that are underserved in existingprogrammes, this study prioritized SGMY with multiple intersecting vulnerabilities; specifically, SGMY who identified as transgender or gender nonconforming (TGD), experienced homelessness, had engagement with the child welfare system, and were immigrants, refugees, or newcomers to Canada.

### Transgender and gender diverse youth

Transgender and gender diverse (TGD) youth have a gender identity that differs from their sex assigned at birth and/or gender expression that eclipses binary male and female categories (Rider et al., 2018). Compared to the general youth population, TGD youth are at increased risk of depression, suicidality, substance use, and self-harm (Rimes et al., 2019; Veale et al., 2017). Compared to cisgender sexual minority youth, TGD youth report more instances of family conflict, bullying, and violence (Choi et al., 2015). TGD youth report hostility from others in public spaces, feeling either invisible or hypervisible to others (Holtby et al., 2015).

### Experiences with homelessness

Approximately 40% of homeless youth identify as SGMY within the USA and Canada (Abramovich, 2016). Homelessness is a dynamic state that changes over time (Castellanos, 2016) due to the concept of home referring to both a physical space and a safe, supportive, secure environment (McCann & Brown, 2018). Adverse childhood experiences or ACEs (e.g., abuse, familial rejection, family conflict) often precipitate homelessness or housing instability (Flentje et al., 2016) and SGMY are disproportionately affected by ACEs (Craig et al., 2020a). Homelessness precipitates psychological and physical distress that for SGMY can be compounded by increased stress concerning their gender and sexuality (Kattari et al., 2017). SGMY experiences of homelessness are heterogenous; for example, trans men are at triple the risk of severe mental and physical health disorders compared to cisgender men due to homelessness (Keuroghlian et al., 2014). It is crucial to examine the intersectional identities of SGMY experiencing homelessness rather than to view each experience independently (Fraser et al., 2019).

### Engagement in child welfare

SGMY are overrepresented in the child welfare system at an approximate ratio of 2.5:1 compared to cisgender, heterosexual youth (Fish et al., 2019). The disproportionality may be even greater as SGMY fear harm if they disclose their gender and sexuality to foster carers and child welfare workers (McCormick et al., 2017). These harms include harassment, physical violence, and sexual abuse (Forge et al., 2018; Woronoff et al., 2006), leading to more frequent placement breakdowns and rehoming, and less likelihood of exiting the child welfare system before reaching adulthood (Fish et al., 2019; Wilson & Kastanis, 2015). SGMY who are removed from abusive home environments by the child welfare system do not experience a decrease in their risk for suicide (Walls et al., 2009). From an intersectional perspective, approximately 50% of SGMY experience homelessness within eighteen months of ageing out of the system (Forge et al., 2018).

### Refugees, immigrants, and newcomers

Although SGMY immigrate to countries such as Canada to escape imprisonment, execution, and victimization, many challenges persist (Logie et al., 2016; Munro et al., 2013). Newcomer SGMY may face homophobia from their families and diaspora communities, as well as racism from white LGBTQ+ people (Alessi et al., 2017; Chiang et al., 2019; Kahn & Alessi, 2017; Lewis, 2016). Further, refugee claimants must repeatedly come out to numerous decision makers and retell their traumatic stories of persecution, which poses risks of re-traumatization (Lee & Brotman, 2013). New SGMY immigrants can feel disconnected from their sexual and gender identities as well as their cultural identities (Fuks et al., 2018). Qualitative research with SGM refugee and asylum seekers has found that they experienced challenging mental health concerns (Alessi et al., 2015) and exhibited resilience. Despite the challenges facing newcomer LGBTQ+ youth, there have also been investigations into the resiliency that youth with multiple marginalization are able to build alongside coping strategies as they grow through these adversities (Chiang et al., 2019; Li et al., 2017).

Given the paucity of research that captures the intersectional experiences of these understudied populations, this study was designed to investigate the intersections of identity and resilience of SGMY through digital photo elicitation methods. Titled QueerVIEW, this study placed priority on participants who identified with one or more of the four priority populations: TGD, experiences with homelessness, engagement in child welfare, and refugee, immigration, or newcomer.

## Methods

QueerVIEW was a digital photo elicitation study utilizing constructivist grounded theory that was conducted in the province of Ontario, Canada from August 2019 to April 2020. QueerVIEW’s study protocol, including data collection tools, has been published elsewhere (Craig et al., 2020b). The study was approved by the University of Toronto’s Health Science Research Ethics Board (Protocol #37041). All methods (including data collection and analysis) were completely digital.

### Photo elicitation

Photo elicitation (e.g., the incorporation of photos into the interview) was utilized. Photo elicitation serves as an alternative to the traditional interview approach as the incorporation of photos provides a stimulus for the elicitation of deeper narratives (Frith & Harcourt, 2007). Harper (2002) suggested that such method is distinct due to the physiological merits of engaging the part of the brain that processes visual information. Photo elicitation helps in rapport building in the researcher-participant dyad context and allows for the bridging of the cultural worlds between them (Meo, 2010). This method is typically participant driven, as participants take or select the photos that guide the interviews, which helps to maintain the focus of the research on the participants and perspectives and responses within their context (Epstein et al., 2006). Photographs can “generate data that illuminate a subject invisible to the researcher but apparent to the interviewee” (Clark-IbáÑez, 2004, p. 1516) and is beneficial when exploring personal aspects of human identity, culture, and community as photographs help to tap into different realms within one’s consciousness (Harper, 2002). Due to its exploratory nature, photo elicitation can foster participant empowerment and creativity as it rejects a structured interviewer led format (Padgett et al., 2013) and allows participants to access emotional responses with more ease. The use of a camera and reflecting on the images have been perceived as an “emotional catharsis” by participants (Padgett et al., 2013, p. 1442). Photos are also not restricted to following a linear or procedural process within the interview which allows for flexibility and spontaneity where the participant can control what photo they discuss and what memories or experiences are associated with it (Lapenta, 2011).

### Recruitment

Purposive and venue-based sampling were employed to recruit participants through a recruitment flyer distributed through the research team’s social media and professional network. Study eligibility included: a) aged 14–29; b) self-identified as SGMY; and c) resided in Ontario, Canada. The flyer then directed participants to an online screening survey hosted by Qualtrics survey software (Qualtrics XM, 2020), which described the study procedures in full for the purpose of informed consent as both a video and written text (McInroy, 2017), confirming that participants had access to technology for capturing photos and participating in a virtual interview, and were comfortable communicating in English. The four priority populations identified above were explicitly mentioned in the recruitment flyer and dyadic questions were posed in the screening survey to confirm identities/experiences. A research coordinator monitored survey responses and, in consultation with the full team, invited participants to the study based on intersecting identification with more than one of the four priority populations. Interview participants received a 25 USD digital gift card in recognition of their time.

### Data collection

Upon being invited to participate, SGMY were instructed to take, select, and submit between 10 and 15 photos via WeTransfer file sharing software. Instructions for photo selection included representation of: a) who participants saw themselves as in their online and offline lives; b) how others may perceive participants both online and offline; c) barriers and challenges to self-expression; and d) what helps participants express themselves and gives them strength despite barriers and challenges. After submitting their photos, participants were invited to attend a semi-structured interview conducted by one of two graduate research assistants (both social workers) trained in photo elicitation interviewing. Interviews were conducted via Zoom web conferencing software and ran from 1.5 to 2 hours. Study procedures were reviewed verbally for the purpose of confirming ongoing consent. The semi-structured interview guide included questions on participants’ personal meanings of intersectionality and resilience, perceptions of online and offline lives, and how they navigate adversity. The “share screen” feature was used by the interviewers to display participant photos and ask probing questions about participants’ feelings and emotions about the image connected to their resilience as SGMY. Interviews were video recorded via Zoom, and the recording was transcribed via natural language processing using NVIVO 12 (QSR International, 2020) and minor transcription errors were subsequently corrected by research assistants.

### Data analysis

Constructivist grounded theory (Charmaz, 2014) was employed by six independent coders who utilized NVIVO 12 data analysis software to simultaneously analyse and integrate interview video (the Zoom interview), transcriptions of the interviews, and submitted photos (10–15) from each participant. Each interview was coded twice by two independent coders who were trained graduate students from ethnically diverse backgrounds identifying primarily as SGMY. Given that constructivist grounded theory situates the researcher as co-constructing meaning and considers context (Charmaz, 2014; Priya, 2019), annotations were used to describe processes occurring in the research (such as interview interruptions, or instances when interview content and nonverbal communication were discordant as well as the researcher’s reactions and interpretations) and captured in the multimodal analytic process designed by the first and second authors in a previous study with SGMY (Craig et al., 2021). Initial coding consisted of systematically identifying codes in vivo based on the photos, interview videos and transcripts (Charmaz, 2014). Focused and axial coding confirmed codes against emerging themes and a model emerged that captures intersectional conception of resilience and identity (Charmaz, 2014). This model was created as a data map using Mindmeister software (Mindmeister, 2020). Data triangulation was designed to support rigour and to capture multiple processes and included side by side coding of the three types of data from each interview (Natow, 2019). To further enhance trustworthiness, each coder maintained an audit trail (Cutcliffe & McKenna, 2004), the research team met four times in three-hour meetings to discuss codes and emergent themes, and member checking was used. An additional consent process was then employed whereby a research coordinator emailed participants individually with photos and quotes that were planned to be used in dissemination and obtained specific consent for use of participant contributions. Member checking was employed in October 2020 whereby seven participants attended a Zoom video call with the researchers in which the emerging themes were discussed, and the data map was reviewed. The remaining 23 participants elected to receive a summary of the findings by email. All participants agreed that the findings reflected their experiences and contributions to the study. Further, participants were given the opportunity to consent to have any of their images featured in an online gallery that is part of the INQYR Rainbow Road living research timeline (https://www.inqyr.org/timeline.html).

## Results

Thirty SGMY completed the study. Participants had an average age of 21.37 and approximately half lived in urban cities with the other half residing in suburban cities. From the four priority populations: a) 60% (n = 18) were immigrants, refugees, or newcomers to Canada; b) 40% (n = 12) identified as TGD; c) 40% (n = 12) had experienced homelessness; and d) 27% (n = 8) had been involved in the child welfare system. 53 per cent (n = 16) of participants identified with two or more priority populations and many disclosed in the interviews that they lived with a mental illness. From the constructivist grounded theory analysis, a model emerged of how participants were working “towards an integrative self” online and offline, with themes of reflecting & knowing, discrimination & intersectional challenges, connecting, performing, curating, coping, (re)defining & (re)creating, and growing & being.

### Towards an integrative self

**Figure 1.**
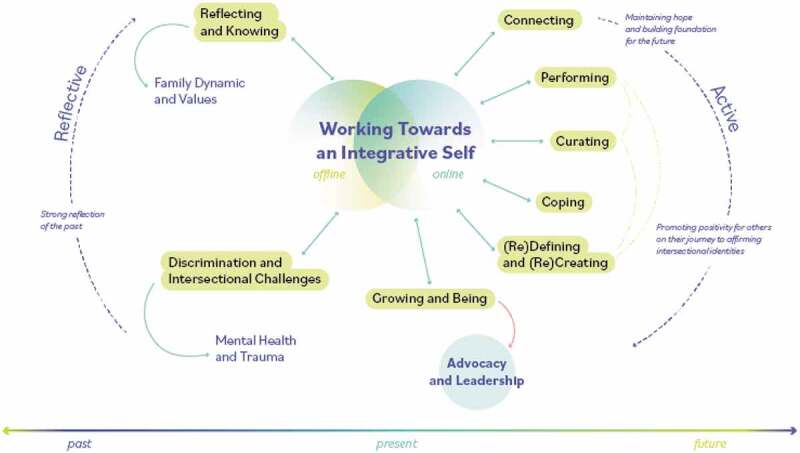
Working towards an integrative self

The process of working “towards an integrative self” emerged as SGMY described various offline and online processes that have contributed to the ways in which they were becoming authentic and “whole” versions of themselves. These processes also assisted youth in embracing their diverse identities in ways that promote their integrative self. Refer to Figure 1 for a visual model of this overarching finding. Gradient colours are used in the figure to denote that findings on the left side were more attributed to past and offline experiences whereas findings on the right side were more focused on the future and online experiences. For each theme in the model, quotes and photos are presented alongside participant demographics. De-identifying (such as blurring faces) was done on participant request.

The following themes emerged and fit in this temporal model that captures the intersection of the past, present, and future and online and offline experiences. To the left of the diagram the data from themes “Discrimination and Intersectional Challenges” and “Reflecting and Knowing” are often described by participants through reflections of the past. This includes past memories and experiences of hardship or trauma. To the right of the model there are themes “Connecting,” “Performing,” “Curating,” “(Re)defining and (Re)creating,” and “Growing and Being.” Working clockwise, the model represents the present and the future as participants begin to harness their resilience and cultivate hope for their future. It is important to note that the model does not represent a linear process for every participant. Rather, it represents a continuous process where participants may be experiencing multiple themes simultaneously as they develop an integrative self. As each theme is introduced, the temporal value of the results will be identified.

#### Theme 1: reflecting and knowing

**Figure 2.**
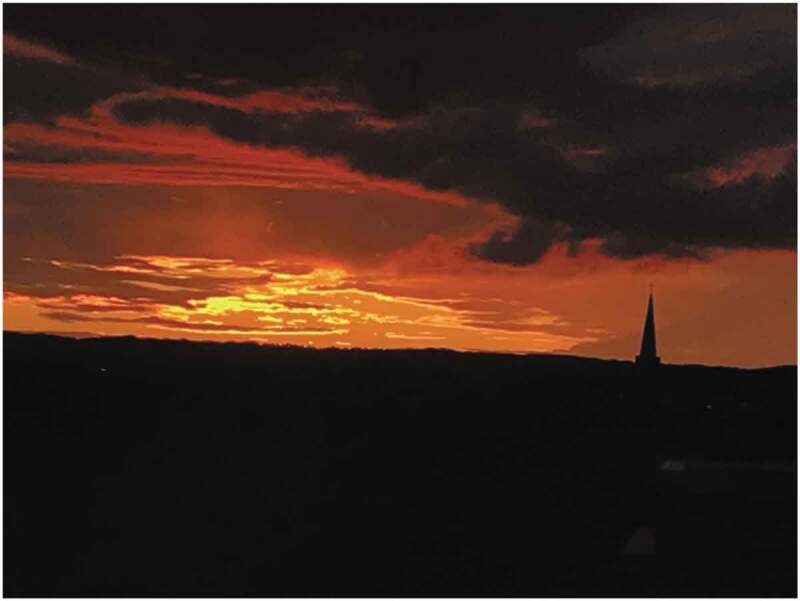
Church in the distance

Reflecting and Knowing serves a resilience mechanism that participants harness in order to move forward after challenging experiences online and offline. This insight and self-awareness show the internal processing that participants go through when they are challenged with adversity. This reflection happened for many youths who reflected upon the mental health challenges and trauma that they had experienced in their lives. This trauma was closely related to the sub-theme Family Dynamics and Values (Figure 1), where youth reflected upon their family’s values and indicated some understanding as to why their values may influence the hardship or rejection they are facing. One participant compared their previous experience with depression to a church (Figure 2) that was positioned in the distance within their photograph.

Oh yeah that’s a church. There’s also a really big church right in front. I don’t know why there’s so many churches close to each other. But I don’t know like maybe that could represent how like my problems are like in the distance because the church is in the distance and with each day that passes, I like move further and further away from the reality that I was in and that I’m in a better place now. (Age 16 Cis woman, lesbian, demi/demi romantic, questioning, homelessness, child welfare)

Another participant discussed their new versus “old” self after moving to Canada as a refugee claimant. As they reflect upon the mental health challenges, anger, and hardship they have faced they also recognize there is a balance of good experiences.

Well I like this me more than I would like the other me, but at the same time this me has much more like mental issues that I never had before, and I’m dealing with issues that I never had to deal with before. I also experienced some very nice feelings that I never experienced before so it’s like good and bad, like I like myself better and I’ve become stronger and more confident but at the same time more hurt, more angry … yeah something like that. But I would still … I like that about myself, I guess. (Age 25 Cis Female, Bisexual, Queer, Newcomer)

Even though some youth describe the challenges in their present day, many of them also use a reflective perspective which shows their strength and resilience mechanisms. There is also analysis of privilege and oppression within this process. Some participants who have strained or conflicting relationships with their family also present an understanding of their parents’ values and cultural differences as in the following quote.

Yes, 100%. Like even if my dad for example, kind of like tunes it out or like is genuinely forgetful or just doesn’t acknowledge that part of my identity, in the end of the day he’s like extremely loving and supportive and I have no doubt in my mind that he will always be there for me even if he’s a little bit confused about it because it’s very - it’s weird to say that he’s old school because he’s not, he’s actually quite modern. It’s just I think he does try to challenge the age-old opinions that he was raised with to understand why we make the decisions that we do in our western lives. He still struggles with that. (TGD, Queer, Homelessness)

#### Theme 2: discrimination and intersectional challenges

Participants described intersectional challenges they faced, which resulted in discrimination in multiple forms. Racism, ableism, homophobia, and transphobia have impacted youth in both online and offline spaces. This caused participants to experience internalized conflict between their own identities and experience challenges with mental health through this trauma (Figure 1). Most participants declined to have photos published per this theme, as the photos frequently referred to family and intimate relationships in their lives. For instance, one youth described that they are proud of their Asian and Queer identities but the conflict that they experience due to their family’s values raises some internal concern and worry about disclosing their Queer identities.

I don’t know being Asian and being Queer, even though it doesn’t feel like wrong to me, it’s just like with my family in a way like my extended family and my dad like the way they’re brought up. They were raised, some people were like raised in different countries and stuff with different ideals and so like it like makes me feel, it makes me feel really terrified to tell them about my Queer identity and so in that way. I feel like it doesn’t fit well, like it doesn’t … it feels normal to me, like I’m proud of it but it’s just in that way it makes me terrified to share that with people in my family that, umm maybe think the way that they do because they were raised with another country with like they’re around that culture and that certain culture. (Age 17, Queer, TGD, Queer)

Sexual and gender identities were part of common intersectional challenges for youth, but their cultural identities also influenced their self-image and the ways in which they felt like they could find connection and value amongst others.

I’m from Bangladesh, I’m Bangladeshi, but I grew up in Canada, I moved here when I was about one or two, so I – my grasp on the language and culture is shaky, and so the other day, I went to – there’s a BSA at my school, Bangladeshi Student Union, and I went. And it was very jarring for me, because everyone was speaking Bangali. Everyone was from like, from Bangladesh. They were raised there and they’re only here for studying. And it was one of those experiences where I felt like I had to leave as soon as I got there. Because I – as much as I don’t fit in with people that aren’t Bangladeshi that can’t understand like where I come from. (Age 20, TGD, Lesbian, Homelessness, Newcomer)

Some participants that live with family members that are unaccepting of their gender and sexual identities often use online platforms to escape the turmoil offline.

Online I can just be more open - just be more about my identity. And you know, like offline version, I kind of have to conform or hide my identity [like with my family]. I just don’t want to deal with the backlash and transphobia. (Age 18, TGD, Queer, Pansexual, Newcomer)

**Figure 3.**
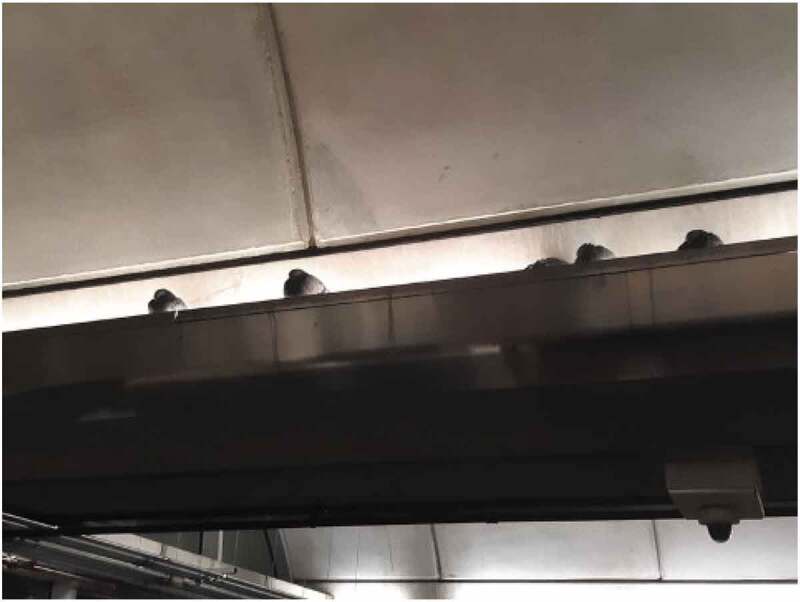
Pigeons

A participant used a photo of pigeons (Figure 3) to discuss their stressors with their family.

I don’t know, [the photo with pigeons] just reminds me of how my relatives, my extended family, treated me. Like they just threw me out, they didn’t care, they didn’t want anything to do with me. And even though I was going through a really turbulent like period in my life they were actually like really happy. Because as far as they knew I was getting what I was - what was coming for me, you know? But I’m still here, still living my best life. (P19, Age 19, TGNC, Lesbian, Homelessness)

#### Theme 3: connecting

**Figure 4.**
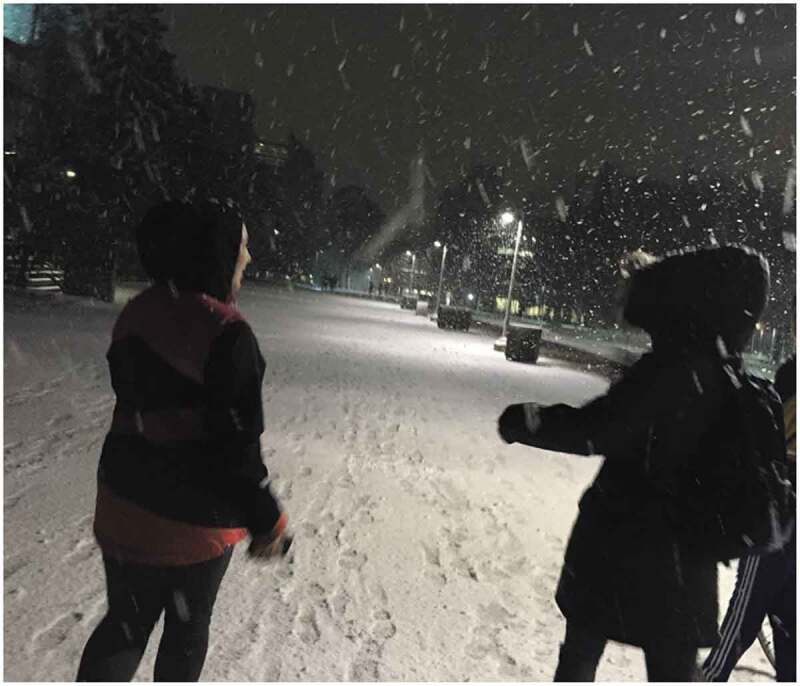
Friend supports

The theme of connecting describes the ways in which participants learn from and collaborate with others. This community can provide safety, support and friendship. Interpersonal connection happens in many external online and offline spaces, influencing an intrapersonal or “internal” process of connecting where participants feel affirmed and increasingly comfortable with their own intersecting identities. External connection is a way for participants to bond with people they might not have access to through online spaces or affirming people external to their family home. Intrapersonal connection often occurs through the ways in which participants utilize coping and self-care mechanisms such as through art, care for animals, or music which increases their self-affirmation and identity development. As one participant said when speaking about technology and developing a support system (Figure 4),

This one is from my first year of university with that group of people who were the first kind of queer and mentally ill people who I had met. We sometimes – we’d go out in the middle of the night to play Pokémon Go because that was when Pokémon go came out. And so we would just like go across the campus collecting Pokémon and stuff … and I still talk with some of them pretty often and some of them not quite as often. And so they they’ve just been like a nice kind of support group for me throughout the years. (Age 21, TGD, Bisexual).

#### Theme 4: performing

Performing is the first theme that is a “present” process in the model (Figure 1). This is a process that youth were commonly engaged with in the present moment. It highlights the way participants utilize the content they learn through online connection or how they perceive popular online content, and outwardly perform this within offline spaces. Education and information sharing offline and then posting images online can also contribute to the theme of performing in offline spaces. Performing has been a way for participants to promote offline connection with others who share similar sexual and gender identities. For example, one participant described the act of using specific items of clothing as a way in which to promote authentic connections with a niche group of people.

I remember I went to the zoo and it was very obviously flagging [non-verbally communicating] as a lesbian. Like I was wearing my flannels and I had a ring of keys on my belt loop and it was just like I was trying very hard to flag because like my friend convinced me. But I remember I went to the zoo and a bunch of volunteers were like oh, you’re flagging, like three gay volunteers, it was awesome. (P19, 19, TGD, lesbian, homelessness)

**Figure 5.**
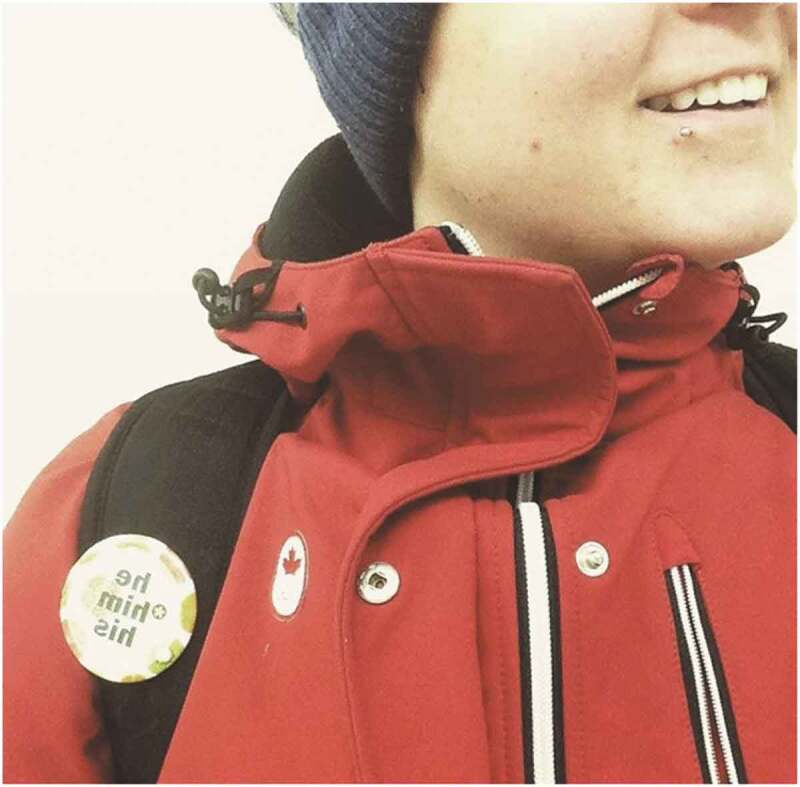
Pronoun pin

Another participant shows a picture of a pin with his pronouns on it as a way to show his appreciation for openly wearing an item that showcases his identity (Figure 5). This picture was taken for followers to get a sense of the pride that the participant feels while wearing the pin.

I got this pin from school … this was when I was just starting my [medical] transition - I thought it was cool to openly wear the pin … I guess trying to show the followers that it kind of looks like I’m proud of my identity, proud of being trans and proud of like these are my pronouns. (Age 24, TGD, Gay)

This further signifies the sentimental value that participants have towards the photographs that they chose for the interview. These photos elicited stories when participants used performing as a way to further explore and develop their identities.

So what I do then is I try to perform. I tried to perform. In the beginning I was trying to kind of impress people kind of to think - to make people think oh … It’s a way of letting people noticing me because I feel like I’m just so lonely and I’m kind … [laughs] I don’t like that word, but I kind of liked attention but not in terms of people want - I want people to hear what I want to say but in terms of I want people to think that I’m cool. So yeah, in that sense, but also this kind of performance kind of emerged into kind of - it’s not really emerging but it’s also mixed with a feeling that I want to know who I am. (Age 18, TGD, Queer, Pansexual, Newcomer)

The theme “Performing” is closely related to “Curating” and “(Re)Defining and (Re)Creating” (Figure 1). While these themes are closely connected due to their relationship to resilience and “present” processes that youth are engaged with online/offline, they are also distinct from each other; this will be further explored.

#### Theme 5: curating

Curating or curation, is a process that participants engage in to promote identity development through various online platforms. Through curation, participants can seek safety, affirm various intersecting identities, and provide a space to build confidence with their identities as they are perceived by others. Participants also acknowledge that they sometimes have an online and offline “persona” that they attempt to maintain in order to control the perception of themselves by others, as shown in the following quote:

I guess there’s only one part that I try my best to come off of online more than offline, and I think I act – I try and post a bit more cooler photos, Online, I think everybody tries it … . Yeah. More gentle side. So, OK, now I. Characterized my online presence as I posted those photos of the cool and happy parts of my life. But that can look intimidating. Yes, I’m posting all these travel photos, all these achievements, friendships, happiness, no vulnerabilities. Which isn’t truthful because it’s like there’s a ton of vulnerability that happens in real life. (Cis female, Queer, Lesbian, child welfare, homelessness)

**Figure 6.**
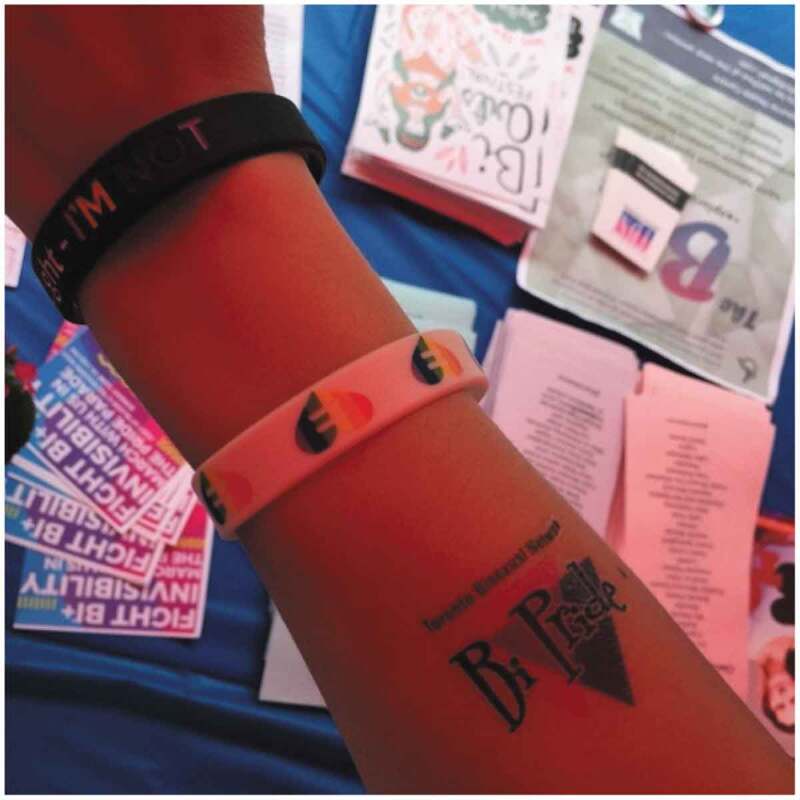
Tattoo and accessories

Curation is a process that also assists participants in becoming selective with the people and supports that they choose to surround themselves with online. Here a participant discusses the importance of this selection as it relates to a tattoo and accessories on their arm (Figure 6) and the person they want to become.

So yeah so those people that I know in real life they are a bit different online. I mean like they all have their own styles or they all post certain things, they all act in certain things, like well for example, someone posts tons of political memes and stuff like that and if I will see it I will know that this is this person … I will only if it’s something super-important. So, it’s just the way you run your own Facebook page for example, you’re the master of it. (Age 25 Cis Female, Bisexual, Queer, Newcomer)

#### Theme 6: coping

Participants often engaged in coping and self-care strategies to mediate daily stressors or larger intersectional challenges and discrimination from others. Some participants found the ability to cope through their love for a pet. Animals were a common element of promoting participant wellbeing. Other participants found moments of solitude in nature as a way to relax and decompress from stress. One participant discussed how meditation helped them to slow down and reconnect.

So I think it was the beginning of high school. I used to go to an arts high school and everyone there was so creative and the energy was so like everywhere and sporadic and my brain was just like everywhere at all times. And I would get home and I’d be so tired all the time. And there was I think I think that was on YouTube. And like someone I followed was like, yeah, I started meditating to just slow down and throw all the garbage out of my brain that I don’t need for the next day. I was like, oh, my God. That was amazing. (Age 18, TGD, Pansexual, Homelessness)

Creative outlets like writing and drawing also assisted participants to cope with challenges that they were facing in online and offline spaces. One participant described their artwork in the following way: “I think though that the looseness of the faces represents like, you know, just like a disconnect between my personality, myself, and my body. I sort of see things that way, loose like that.” (Age 21, TGD, Bisexual, Homelessness, Child welfare).

**Figure 7.**
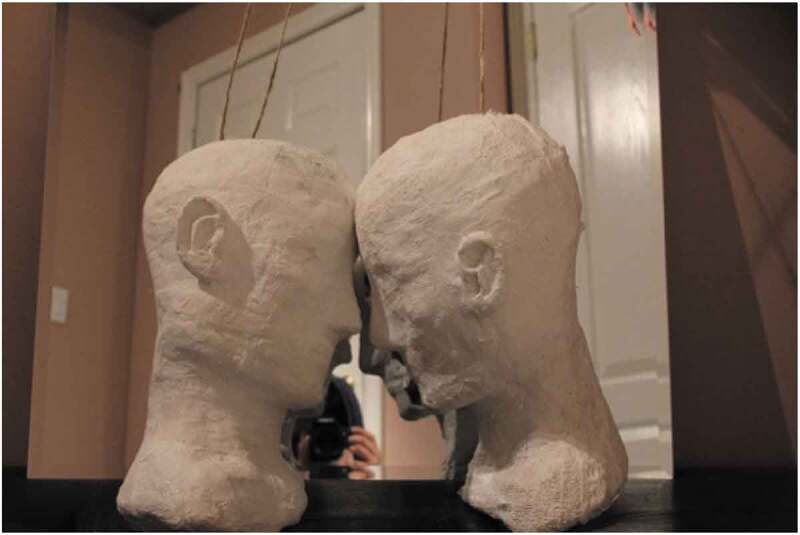
Sculpture

Another participant described the art they created (Figure 7) as a representation of the way that they have coped with feelings of anxiety, jealousy, and the desire to escape from themselves through others. This quote also discusses a selfie taken with a group of people in a mirror, that the participant declined to have disseminated.

A lot of the times I use like my relationships with other people that helped me cope with like difficulties and that I’m going through. And so the idea is like I use like other people to escape, umm, to escape like negative things … it’s blank on the front but I’ve struggled with like the turtles, the weight and like the person on the left is like how I feel with my anxiety sometimes and on the right is like is like representing my jealousy and like always seeing other people and wanting to be like them and seeing everyone happy … and like also people telling me to smile or try to be happy and stuff which is what you can see because of the mirror on the back, so it’s also like your it looks like the people are like escaping from you which is it’s like another idea in my piece that I don’t know if that makes sense. (Age 17, Queer, TGD)

**Figure 8.**
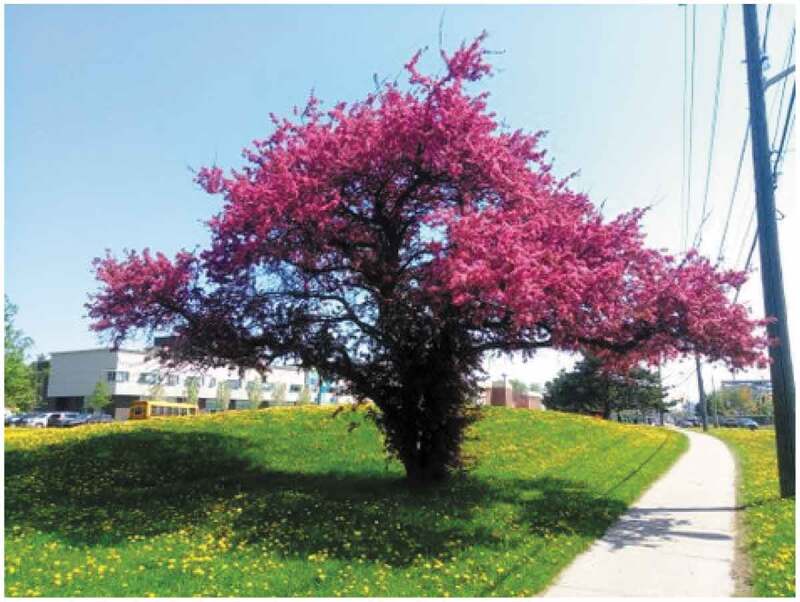
Tree

One more participant described how engaging with nature (Figure 8) helped them cope.

If I’m just going on a walk along the trail and I know I’m not going to be running into people, I am much less concerned about how people are going to see me … If I want to wear, you know, whatever thing today, I’m going to do that. And like the actually the photo there … that one I enjoyed because that there are the little flowers there, too. That is on the walk between my house and the subway station. And it is near a high school where I’ve been heckled by teens a few times. But then it’s just like – here is this big, beautiful pink tree and all of these flowers. And just like it makes me happy to see just like beautiful things along the everyday life. (Age 23, TGNC, Queer, Lesbian, Queer).

#### Theme 7: (Re)Defining & (Re)Creating

As participants become increasingly secure through curation of their online world, they then engage in “(Re)Defining and “(Re)Creating” of their offline spaces in order to live as authentically as possible while maintaining safety. Some participants have online experiences that influence the way they recreate their lived experiences offline. This process of redefining one’s offline experience can promote wellbeing and confidence in participant identities. Youth also created spaces for themselves and others that they did not see represented after finding inspiration from Queer advocates and social media influences online.

For instance, one participant described their increasing confidence with identifying as non-binary in their offline life because they learned more from other non-binary people through social media. This realization allowed them to understand that they could maintain their own definition of what it means to be non-binary and not conform to a perceived image of what it means to have this identity.

You can be like more than just online. And then about a year ago in New Zealand I met someone who is non-binary. And so I was like OK so like you can actually do that in real life then. And so I kind of like I have become very confident in saying that now. But I just don’t like having to defend myself about it and like explaining to just like any random person like oh no actually I’m non-binary and we’ll be like oh so like you do X thing. And then I’m like oh no actually it means this that it’s got these like kind of layers to it and it’s different for everyone. (Age 21, TGD, Bisexual)

Other participants redefine their offline life in ways that promote safety while affirming their identities. One participant described the way they used clothing to promote their identity while avoiding confrontation or conflict with their family members. This participant’s family would not allow them to dress in “feminine” clothing. As a result, the participant redefined what clothing they could wear by accepting gender neutral pieces in order to appease family and, more importantly, feel more secure in their gender identity.

Yeah. Yes, you have something I [have a favourite shirt]. I’m wearing the same one today. It’s just like ahh I kind of like that sweater. It’s kind of gender neutral, so – It’s not like explicitly male or female so it kind of – I guess it kind of confirms my gender identity. (Age 18, TGD, Queer, Pansexual, Newcomer).

**Figure 9.**
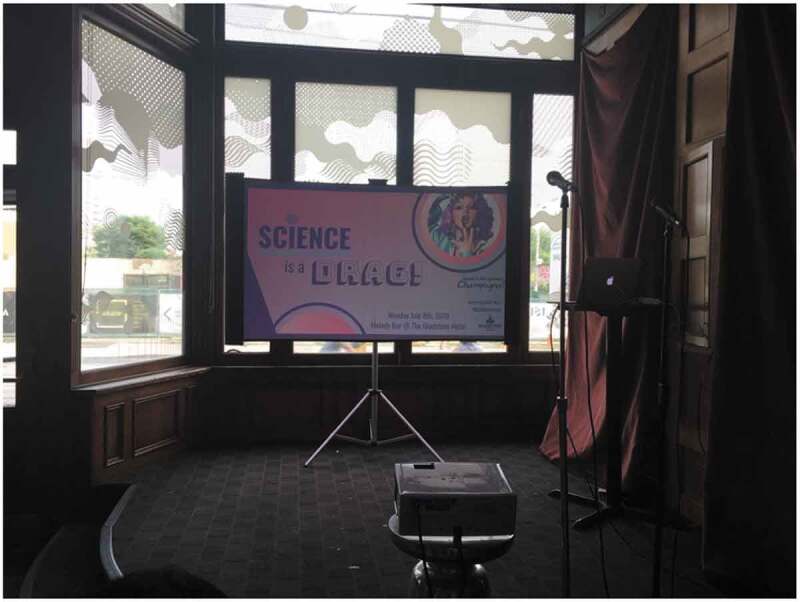
Science is a drag event

Another participant hosted events and created spaces offline, not only for themselves, but to reach other Queer students with similar intersectional identities within science (Figure 9).

But so they were like, yeah. It’s so great to see someone in a position of leadership on LGBT men who like East Asian and like holding like this position is play such a big leadership position like speaking on stage in front of everyone and organizing this event, and it means a lot that I wasn’t expecting that message. I think a lot of times I talk about my career and trends on Twitter, but I forget that I’m also a racialized person and like – I don’t forget. And it means a lot to me. It’s important to me but I forget that like – there are people who are like me in that they’re also queer and trans and East Asian and oh, well, all these things and you see someone who’s so closely like mirrors, their personality is such an impactful thing. (Non-binary, Genderqueer, Bisexual, Queer, Pansexual, Newcomer)

(Re)Defining and (Re)Creating can also be creating an experience where participants feel validated and affirmed in various interactions with strangers or their family and friends. For example, one participant discussed the importance of stating their name to an employee at a coffee shop.

So I went up to the Starbucks employee. They’ve never met me in their life. I can just say my name is “name” and they can just be like, cool. But they don’t think about it. They don’t think, hey, didn’t you used to be “name”? So things like that. Like online submissions, like talking to new people or like people who I know I won’t see you again. It’s like, you know, it’s nice when you say that. (Age 15, TGD, Queer, Bisexual, pansexual, Queer).

#### Theme 8: growing up & being

The theme Growing Up & Being describes the way participants perceived their growth process and the ways in which their own growth, resilience, and perseverance translate to helping others in online and offline spaces with similar intersectional identities. Growing up and being was closely connected to advocacy and leadership among many participants. Some participants explained that as they were growing and feeling more comfortable living with multiple intersectional identities, they posted more pictures and made more posts on different social media platforms to support others. These public or private accounts promoted inclusivity and validation of identities for others who may have been experiencing similar challenges. Many participants explained that they wanted to make themselves more visible, even if they were using anonymous accounts, to provide representation for others that they did not see online.

It was like more for like the audience to let people know, like you know, because there’s a lot of people out there who has their own situation, their own struggles. So, it’s like OK guys like you’re not the only person that’s going through something. So, like it’s OK to smile so you know don’t worry like life is not over. Just continue to do what you’re doing. So, it’s like an uplifting type of thing to other people who are actually going through a hard time in their life too. (Age 29, TGD, Queer, Lesbian, Queer, Homelessness, Child Welfare)

**Figure 10.**
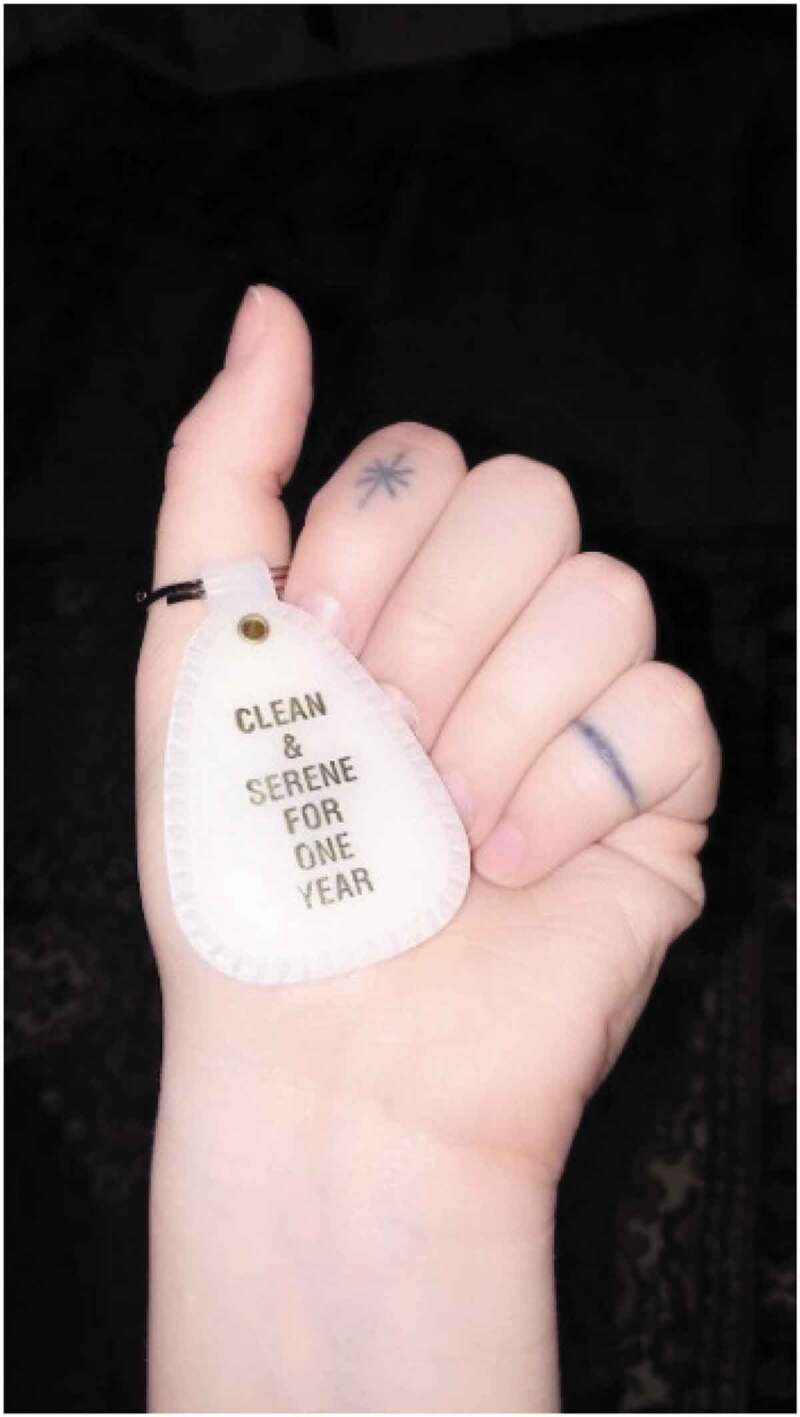
12-step anniversary token

Advocacy also extends to offline spaces, such as addiction and mental health recovery programmes (Figure 10) where participants feel comfortable supporting others who have similar life experiences and intersectional identities.

Being in a 12 Step program it’s all about what they call sponsorship, which is essentially just, like an older sibling leading you down … someone who’s been doing this longer and someone just saying this is how I did it, and you can take their help or not take their help. But I learned that a lot of things in my life I cannot do alone, and not that I’m necessarily going to be the person to help everyone else, but if I can have someone who unpacked everything, like all the time, like if I didn’t see that one thing or if I didn’t hear this … and I have a sponsor and I sponsor. I sponsor two people in my program, and I need to lead by example. I cannot expect them to take my suggestions if I’m still hiding. Like, this is … I’ve never had to get so vulnerable. (Age 20, Cis female, Queer, Bisexual, Pansexual, Homelessness, Child Welfare).

## Discussion

This study illuminated the various ways in which four key populations of SGMY navigated the intersections of their identities. Specifically, this study prioritized participation from SGMY who: a) were trans and gender diverse; b) had experienced homelessness; c) had engaged in the child welfare system; and d) were newcomers, immigrants, and refugees. Their insights contributed to a model of working towards their integrative selves both online and offline. This involved accepting their intersectional identities while they were also growing and evolving. Online communities provided space to evolve and grow in a safe way where these identities and feelings of belonging could be translated offline. For example, participants took control of their online content and actively pursued positive experiences. This behaviour relates to previous research about SGMY utilizing adaptive coping strategies to avoid negativity (Craig et al., 2019).

### Intersectional narratives of resilience

This study may be the first qualitative project to focus on these four priority populations within SGMY. While studies have been conducted with SGMY who are trans and gender diverse (Rimes et al., 2019), homeless (Flentje et al., 2016), in the child welfare system (Forge et al., 2018), and immigrants, newcomers, and refugees (Alessi et al., 2017), this study offers new insight at the intersection of these vulnerable identifies and experiences. Participants actively explored their own experiences of resilience as they reflected upon their past and the challenges they faced internally and externally. Importantly, study participants had a range of traumatic experiences in families and systems that had significant long-term impacts on their mental health and wellbeing. Developing an understanding of how they navigated those intersecting challenges, such as a history of child welfare involvement through multiple foster carers compounded by housing instability, can enable more prepared systems. However, participants consistently complemented their challenges with the privileges they held within the same stories of pain and rejection. These privileges not only included identities, such as being trans and gender diverse and navigating a gender identity and presentation that differed from their sex assigned at birth, but the communities that participants were able to connect with online and offline, such as those formed during the immigration process or focused on SGMY and their interests. This is consistent with what Meyer (2003) refers to as group or community coping (Meyer, 2003; Sterzing et al., 2017). Importantly, youth maintained that finding communities of SGMY who also shared their cultural, ethnic, and religious identities were valuable and sharing a singular identity such as gender was not always fulfiling and conducive to building their sense of self. Ultimately, the insights from this study uncover facilitators and barriers to SGMY’s efforts to become a truer version of themselves both online and offline.

### Digital photo elicitation

Digital photo elicitation was an appropriate method for a study of SGMY intersectionality and resilience as it allowed youth to have a stronger voice in studies of their experience and to direct the research process while examining the context of their lives (Liebenberg et al., 2014). The digital photo elicitation methods provided access to a range of SGMY, including those with mental health struggles, and provided an opportunity for youth to manage their own data and research process. For example, many participants chose photographs that they had previously saved on their personal electronic devices as they had stated that some of these photos had been posted on their social media accounts. This also highlights the temporal value of the research as it showcases how the past has been interwoven with stories of the present and hopes for the future. Pictures from the past can simultaneously represent what they have been through, how they have grown and what they hope for in the future. When analysing the selected photographs, it was evident that they were not exclusively related to online or offline experiences. Many selfies or “planned” pictures also seemed to reflect aspects of both online and offline identities as participant stories unravelled within the interviews. For example, some participants chose selfies or photographs that were important and sentimental to reflect their offline lives that fit well with the theme “Connecting.” These visuals showcased items or activities (e.g., artwork, animals and pets, nature, plants, games cosplay) that helped them in their lives as they were experiencing discrimination and intersectional challenges. Creative and calming outlets that supported their coping were common among many participants.

### Limitations

Study limitations include the importance of youth access to technology in order to share their photos and participate in a virtual interview. This could have prevented youth with experiences of homelessness and lack of access to technology and privacy to participate. The interviews were also conducted in English as there were not any resources available to conduct interviews or analyse data in another language. This may have been a barrier for youth who are newcomers, immigrants, and refugees that speak a language other than English. Potential participants in early 2020 may have been impacted by the COVID-19 pandemic because there was not an opportunity to interview participants who preferred to interview in person. This could have also had implications concerning the privacy of SGMY, for instance, when doing a virtual interview requires being in a space shared lived with unsupportive family members or roommates. Future studies may consider collecting demographics regarding ethnicity, relationship status, immigration age, and prior countries of residence to further illuminate distinct participant narratives and explore the influence of other risk factors, such as personal and family religiosity, on SGMY wellbeing.

## Conclusion

The use of digital photo elicitation methods through this study that prioritized participation from SGMY with multiply marginalized experiences and identities resulted in a complex understanding of how they navigate their online and offline environments, and actively work towards a more authentic and integrated sense of self. SGMY’s vibrant contributions allowed for a temporal model of their intersectional resilience to be constructed that provides insight and nuance into their strengths and challenges and can provide guidance for future research and practice.
